# Homoploid hybridization of plants in the Hengduan mountains region

**DOI:** 10.1002/ece3.5393

**Published:** 2019-06-23

**Authors:** Rui Yang, Ryan Folk, Ningning Zhang, Xun Gong

**Affiliations:** ^1^ Key Laboratory for Plant Diversity and Biogeography of East Asia Kunming Institute of Botany, Chinese Academy of Sciences Kunming China; ^2^ Key Laboratory of Economic Plants and Biotechnology Kunming Institute of Botany, Chinese Academy of Sciences Kunming China; ^3^ University of Chinese Academy of Sciences Beijing China; ^4^ Florida Museum of Natural History University of Florida Gainesville Florida USA; ^5^ Yunnan Key Laboratory for Wild Plant Resources Kunming China

**Keywords:** Hengduan mountains region, homoploid, hybrid zones, natural hybridization, reproductive isolation

## Abstract

The Hengduan Mountains Region (HMR) is a major global biodiversity hotspot. Complex tectonic and historical climatic conditions created opportunities for natural interspecific hybridization. Likewise, anthropogenic disturbance potentially raises the frequency of hybridization. Among species studies to date, the frequency of homoploid hybridization appears in the HMR. Of nine taxa in which natural hybridization has been detected, three groups are involved in homoploid hybrid speciation, and species pairs from the remaining six genera suggest that continuous gene flow occurs in hybrid zones. Reproductive isolation may greatly affect the dynamic and architecture of hybrid zones in the HMR. Asymmetrical hybridization and introgression can primarily be attributed to both prezygotic and postzygotic barriers. The frequent observation of such asymmetry may imply that reproductive barrier contributes to maintaining species boundaries in the alpine region. Ecological isolations with environmental disturbance may promote breeding barriers between parental species and hybrids. Hybrid zones may be an important phase for homoploid hybrid speciation. Hybrid zones potentially provided abundant genetic resources for the diversification of the HMR flora. The ecological and molecular mechanisms of control and mediation for natural hybridization will help biologists to understand the formation of biodiversity in the HMR. More researches from ecological and molecular aspects were required in future studies.

## 1 INTRODUCTION

The Qinghai‐Tibetan Plateau (QTP) and its adjacent Hengduan Mountains Region (HMR) have been considered as one of the important biodiversity hotspots in the world (Myers, Mittermeier, Mittermeier, Da Fonseca, & Kent, 2000). The HMR in particular harbors the richest temperate flora of seed plants in the world and is considered to be among the areas with the high concentration of endemic species in the world (Li & Li, 1993; Wu, 1988). The region comprises about 16,550 species, which consist of 2,264 genera and 227 families (3,300 endemic species and 90 endemic genera) (Sun, Zhang, Deng, & Boufford, 2017). In the alpine zone of the HMR, the number of seed plant species is two to three times than that in other known alpine region (Xu, Li, & Sun, 2014a, 2014b).

The HMR is located at the eastern end of the Himalayan regions and the south‐eastern boundary of the Qinghai‐Tibet Plateau (Li, 1987; Sun et al., 2017). Climate shift accompanied by orogenic events may lead to geographical overlap of some species, which impel the frequent contact of plants in the HMR. The rate of species differentiation may coincide with the rapid uplift of the HMR, and the latter may have facilitated the diversification of species in the late Miocene (11.6–5.3 MYA) (Sun et al., 2017; Xing & Ree, 2017).

Multiple mechanisms, such as allopatric speciation via geographical isolation, natural hybridization, and allopolyploidy, are thought to promote species diversity in the HMR (Liu, Duan, Hao, Ge, & Sun, 2014; Liu, Wang, Wang, Hideaki, & Abbott, 2006; Nie, Wen, Gu, Boufford, & Sun, 2005; Wen, Zhang, Nie, Zhong, & Sun, 2014; Xing & Ree, 2017). Of these mechanisms, natural hybridization may be an important generator of plant diversity in the HMR. Natural hybridization has long been considered as an important evolutionary phenomenon in plants, especially in flowering plants, and its potential role in the origin of species has been discussed since the time of Linnaeus (Baack & Rieseberg, 2007; Larson, 1968; Stebbins, 1959). According to one recent investigation, natural hybridization is involved in 25% of plant species (Mallet, 2005; but see Folk, Soltis, Soltis, & Guralnick, 2018). Furthermore, natural hybridization can facilitate speciation and innovation through transferring adaptive traits via introgression, formation of recombinant forms, or allopolyploidization (Abbott, Hegarty, Hiscock, & Brennan, 2010; Mallet, 2007; Rieseberg & Carney, 1998; Soltis & Soltis, 2009). In contrast to allopolyploidy, homoploid hybridization is characterized by hybridization between parental species without a change in chromosome number and result in the formation of novel hybrid species or hybrid zones (Abbott et al., 2013; Gross & Rieseberg, 2005). Homoploid hybridization between species continually produces hybrids of mixed ancestry in hybrid zones (Harrison, 1993; Payseur, 2010). However, in hybrid zones, hybrids do not instantly establish as a new species need time to produce isolation barriers with their parental species. Hybrid individuals consist of early‐ or later‐generation hybrids in hybrid zones. In some cases, hybrid swarm (various recombinant types) is found in hybrid zones (Abbott, 2017; Barton & Hewitt, 1985). The location of hybrid zones is often either only a few hundred meters wide or may be several hundred kilometers long (Barton & Hewitt, 1985). For homoploid hybrid speciation, the origin of a novel lineage reproductively isolated from its parents by ecological and spatial barriers after formation of hybrids between parental species in hybrid zones (Buerkle, Morris, Asmussen, & Rieseberg, 2000; Gross & Rieseberg, 2005; Rieseberg, 1997). Hence, hybrid zones may be an important form for homoploid hybridization until hybrids are established.

Although on the basis of recent surveys (Soltis, Visger, & Soltis, 2014; Soltis & Soltis, 2009; Yakimowski & Rieseberg, 2014), homoploid hybrid speciation appears to be less common than allopolyploid speciation, homoploid hybridization can be potential resources for plant evolution (Soltis & Soltis, 2009; Stebbins, 1959). However, at the regional scale, cytological statistical analysis of the chromosome numbers of 552 taxa of native angiosperms in the HMR suggest polyploidy may only play a minor role in the evolutionary diversification of the region (Nie et al., 2005). This conclusion has been supported by other investigations (Chen et al., 2014; Liu, 2004; Liu, Liu, Ho, & Lu, 2001; Liu, Zhou, Ho, & Lu, 1999). Therefore, homoploid hybridization may be a common type of natural hybridization in the HMR. In the region, cases of hybrid speciation have been reported to date at the diploid level, that is, *Pinus densata*, *Picea purpurea*, and *Ostryopsis intermedia* (Lu, Tian, Liu, Yang, & Liu, 2014; Sun et al., 2014; Wang, Szmidt, & Savolainen, 2001). Additionally, more genera in the HMR are involved in naturally occurring homoploid hybridization within hybrid zones (Table S1), such as *Ligularia* (Pan, Shi, Gong, & Kuroda, 2008; Yu Kuroda, & Gong, 2011, 2014; Yu, Pan, Pan, & Gong, 2014; Zhang, Yu, Wang, & Gong, 2018; Zhang, Gong, & Ryan, 2017), *Rhododendron* (Ma, Milne, Zhang, & Yang, 2010; Marczewski, Chamberlain, & Milne, 2015; Yan, Gao, & Li, 2013; Zha, Milne, & Sun, 2008; Zhang, Zhang, Gao, Yang, & Li, 2007), *Primula* (Ma, Tian, Zhang, Wu, & Sun, 2014), *Roscoea* (Du, Zhang, & Li, 2012), *Silene* (Zhang, Montgomery, & Huang, 2016), and *Salix* (Wu, Wang, Yang, & Chen, 2015).

The objectives of this paper are to (a) discuss the potential factors which trigger natural hybridization in the alpine region; (b) briefly review natural hybridization and homoploid hybrid speciation in the HMR; (c) discuss the effects of reproductive isolation on the level and direction of gene flow in hybrid zones; and (d) propose how natural hybridization may be important to the diversification of the HMR flora.

## 2 FACTORS WHICH TRIGGER HYBRIDIZATION IN THE HMR

### 2.1 Tectonic and climatic changes in the history

**Figure 1:**
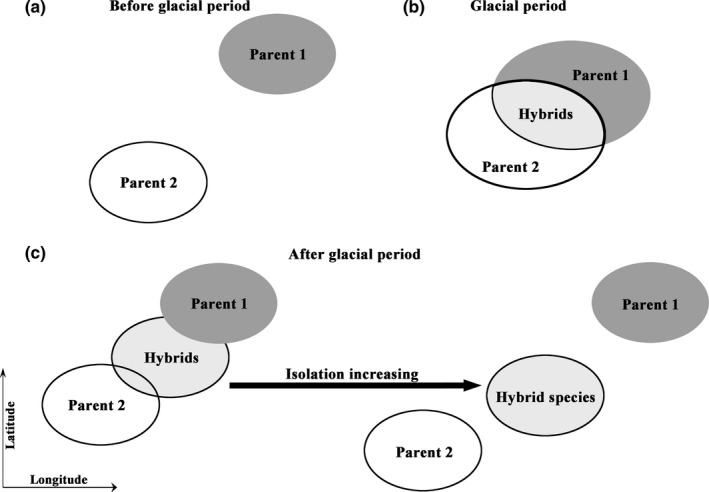
The landscape of homoploid hybrid speciation with geographical and glacial changes in the HMR. (a) Parental species are allopatric distribution before glacial period; (b) Both parents are contacted in sympatric site during glacial period; (c) Homoploid hybrid species are differentiated from their parents by isolation increasing

Historical–geographical processes and/or climate change may trigger rapid speciation in the mountains region (Liu, Duan, et al., 2014; Liu et al., 2006; Sun et al., 2017; Xing & Ree, 2017). Moreover, these historical events, especially for the uplift of the HMR, may also have offered opportunities for related species to contact (Anderson & Stebbins, 1954; Folk et al., 2018; Frenzel, 1968; Liu, Duan, et al., 2014). Secondary contact in parental species may be caused by geographical history with climate change (Abbott, 2017; Folk et al., 2018; Liu, Duan, et al., 2014; Sun et al., 2017). Three potential homoploid hybrid species are known from the HMR, and two of them suggest hybridization may be associated with orogenic and climatic changes in the past of the HMR (Figure 1). *Picea purpurea* is thought to have originated via homoploid hybrid speciation between *Picea likiangensis* and *Picea wilsonii* at the Pleistocene (Sun et al., 2014). An analysis of evolutionary history shows that during the Quaternary glaciation, climate shift may facilitate contact between both parents (Figure 1b), which result to trigger the hybrid origin of *P. purpurea* (Sun et al., 2014). In addition, *O. intermedia* is a diploid species of hybrid origin, deriving from hybridization between *Ostryopsis davidiana* and *Ostryopsis nobilis* (Liu, Abbott, Lu, Tian, & Liu, 2014; Lu et al., 2014). *Ostryopsis davidiana* may have migrated southward and made range contact with *O. nobilis* during a glacial maximum, creating a historical opportunity for hybridization that does not exist in the present due to allopatry among the parents (Figure 1 and Table S1).

### 2.2 The effect of anthropogenic disturbance

Environmental disturbance is regarded as a significant factor that facilitates hybridization (Abbott et al., 2013; Anderson, 1948, 1949; Anderson & Stebbins, 1954; Harrison & Larson, 2014; Rieseberg & Carney, 1998; Thomas, 2015). In the case of *Iris* from the Mississippi delta, differences in prevalence of hybrids and of hybrid derivative classes are affected by agricultural activity and hybrid region is limited at the border of the farm (Riley, 1938). Thus, anthropogenic disturbance has been thought to generate opportunities for hybridization and novel habitat or hybridized habitats for the persistence of hybrids (Anderson, 1948; Anderson & Stebbins, 1954; Guo, 2014). Besides, secondary contact of diverged species, due to habitat disturbance, is thought to promote the formation of hybrid zones (Abbott, 2017). A handful of cases to date revealed that anthropogenic disturbance potentially promotes hybridization by extending and altering plant phenology, especially for flowering time, creating opportunities for species to exchange genes that otherwise would not under undisturbed conditions (Ellstrand & Schierenbeck, 2000; Lamont, He, Enright, Krauss, & Miller, 2003; Meerow, Gideon, Kuhn, Mopper, & Nakamura, 2011; Ortego, Gugger, & Sork, 2017; Vallejo‐Marín & Hiscock, 2016; Yakimowski & Rieseberg, 2014).

In case studies to date, it has frequently been inferred that the formation and composition of hybrid zones are potentially associated with anthropogenic disturbance in the HMR (Ma, Tian, et al., 2014; Wu et al., 2015; Yu, Pan, et al., 2014; Zha, Milne, & Sun, 2008, 2009; Zhang et al., 2018; Zheng et al., 2017). Distinct hybrid derivatives are known that may have been arisen from human disturbance in *Rhododendron* (Table S1). In two hybrid zones of *Rhododendron irroratum* and *Rhododendron delavayi* studied with chloroplast markers, gene flow is unidirectional and F_1_s dominate in one, whereas gene flow is bidirectional and F_1_s occurs with other classes in another site that experienced human disturbance (Zha, Milne, & Sun, 2009). Higher hybrid population frequencies and sizes occur in hybrid zones between *R. decorum* and *R. delavayi* in disturbed habitats (Zha et al., 2008). Long isolation and enough mutations may lead to *Ligularia* sympatric species coexist without hybridization (Liu et al., 2006; Zhang et al., 2018). However, pre‐existing isolation barrier may have been broken via human disturbance. Thus, both hybrid groups from three sympatric *Ligularia* have been formed in an area subject to human disturbance (*L. cyathiceps* × *L. duciformis* and *L. duciformis* × *L. yunnanensis*) (Zhang et al., 2018). Moreover, in three further species pairs (*Ligularia*, *Primula*, and *Salix*), it has been inferred that habitat disturbance may result in different patterns of hybrid classes in zones of sympatry (Ma, Tian, et al., 2014; Ma, Xie, et al., 2014; Wu et al., 2015; Xie et al., 2017; Yu, Pan, et al., 2014). Although these case studies have allowed us to make preliminary conclusions about the effect of anthropogenic activities on natural hybridization, the relationship between hybridization and environmental disturbance requires future work.

Different types and degrees of disturbance (natural and/or anthropogenic) are considered important in the formation of hybrid zones (Abbott, 2017). We found only one case that tried to analyze and distinguish these differences: Wild fire frequency but not human activities have been found to increase hybridization between *Quercus berberidifolia* and *Quercus durata* (Abbott, 2017; Ortego et al., 2017). No work of this kind has been implemented in the HMR; future work is needed to understand the types of disturbance that may have led to present‐day hybrid patterns.

## 3 HOMOPLOID HYBRID SPECIATION IN THE HMR

Recent reviews on homoploid hybrid speciation have identified more than 30 well‐characterized cases of this phenomenon in the plant kingdom to date. (Feliner et al., 2017; Schumer, Rosenthal, & Andolfatto, 2014). But the importance of homoploid hybrid speciation may be severely underestimated in these identifications by excessively stringent criteria and by ignoring the effect of timescales (Folk et al., 2018; Feliner et al., 2017). Two classic examples are found in North American *Helianthus* and *Iris* (Arnold, 1993; Rieseberg, Van Fossen, & Desrochers, 1995). Ecological isolation is an important component of reproductive barriers in homoploid hybrid species (Abbott et al., 2013; Gross & Rieseberg, 2005; Taylor, Willard, Shaw, Dobson, & Martin, 2011). Hence, homoploid hybrid species generally occur in habitats which are isolated from their parental species (Rieseberg, 1997). Hybridization can act as a force to recombine pre‐existing genetic variation in novel ways. This is a potential mechanism allowing hybrids to spatially and ecologically diverge and potentially colonize niches unexploited by their parental taxa (Buerkle et al., 2000; Mallet, 2007). As described above, homoploid hybrid species are generally originated from parental sympatric region. Ancient hybrid zones may be arisen in the region before ecological niche differentiation between parents and hybrid lineages. Drastic tectonic and climatic changes in the history of the HMR may provide new ecological niches and facilitate completely habitats divergence between hybrid species and its parents (Liu, Duan, et al., 2014; Wen et al., 2014; Xing & Ree, 2017).

*Pinus densata* is a species that originated from hybridization between *Pinus tabuliformis* and *Pinus yunnanensis* (Gao et al., 2012; Ma, Zhao, et al., 2010; Ma, Szmidt, & Wang, 2006; Song, Wang, Wang, Ding, & Hong, 2003; Song et al., 2002; Wang, Mao, Gao, Zhao, & Wang, 2011; Wang & Szmidt, 1990; Wang et al., 2001). Previous studies have suggested that ancient hybrid zones occurred in overlapping region of both parental species, with the exception of the contemporary allopatric/partly sympatric distributions of *P. tabuliformis* and *P. yunnanensis* (Wang et al., 2001). Gene flow may have occurred between ancestrally sympatric populations that were subsequently separated by the uplift of the HMR (Gao et al., 2012; Song et al., 2003). A series of studies suggest that the origin of *P. densata* is estimated to be late Miocene, a timing that coincides with recent major geological events in the HMR (Gao et al., 2012). Homoploid hybrid species, *P. densata*, today mainly occurs at higher elevational zones than those occupied by either parental species (Song et al., 2003; Wang et al., 2011, 2001). Additionally, *P. densata* can survive in water‐limited high‐elevation habitats due to having evolved several physiological traits that are adapted to extreme habitats in the HMR (Ma, Zhao, et al., 2010). Hence, the ecological and geographical differentiation is associated with the isolation between *P. densata* and its parents (Table S1).

Similarly, with the species pair *P. wilsonii* and *P. likiangensis*, ecological isolation may potentially have created reproductive barriers between homoploid hybrid species (*P. purpurea*) and these two parental taxa (Sun et al., 2014). Based on molecular data, *P. purpurea* not only shares alleles with both parents, but also possesses more unique alleles relative to alleles shared with either parent. These unique alleles may be relative to adapt new habitat in hybrid species. Nuclear data indicate that the origin of *P. purpurea* occurred at approximately 1.3 MYA; during this period, climate shift may lead to range contact between *P. wilsonii* and *P. likiangensis* (Sun et al., 2014). Thus, historical climate change may have contributed to hybridization between *P. wilsonii* and *P. likiangensis*. Currently, *P. purpurea* is isolated ecologically from its parents via occupying higher elevation. Demographic modeling results indicate that *P. purpurea* experienced geographical range expansion about 0.75 MYA, while both parents were inferred to have returned to their former regions during this period (Sun et al., 2014). Alpine areas after the glaciation have been inferred that provide available regions for geographical expansion of *P. purpurea* (Sun et al., 2014). Similar geographical expansions have been reported from another hybrid species in the HMR, *P. densata* (Gao et al., 2012). Several studies of other taxa show such expansions of geographical range in mountainous areas and adjacent regions (Li et al., 2012; Liu et al., 2013; Liu, Sun, Ge, Gao, & Qiu, 2012; Sun, Ikeda, Wang, & Liu, 2010; Wu, Cui, Milne, Sun, & Liu, 2010).

Likewise, climatic oscillations in the Quaternary may also have spurred hybridization between *O. davidiana* and *O. nobilis*, potentially triggering the homoploid hybrid origin of *O. intermedia* (Liu, Duan, et al., 2014; Lu et al., 2014). On the basis of cpDNA data, *O. intermedia* is closely related to *O. nobilis*, whereas, based on nuclear data, *O. davidiana* mainly contributes to the nuclear composition of *O. intermedia*. A combination of ecological niche modeling and paleoclimatic data for the last glacial maximum revealed that the parental taxa experienced historical rang overlap during glacial maximum conditions (Figure 1b). Subsequently, *O. davidiana* retreated to northern China during subsequent climatic warming, which may have reduced competition with *O. intermedia* (Figure 1c). In addition, *O. intermedia* expanded its distributional range to new niche space unoccupied by *O. nobilis*, which may have led to the fixation of the single observed haplotype in hybrid lineage (Lu et al., 2014). Based on these results, it appears that the origin and isolation of *O. intermedia* from its parents may potentially have resulted from historical climate change (Liu, Abbott, et al., 2014; Lu et al., 2014).

In summary, environmental heterogeneity associated with the uplift of the HMR and subsequent historical climate dynamics may have promoted rapid speciation in this region (Liu et al., 2012, 2006; Qiu, Fu, & Comes, 2011). Major changes in topography and climate may have been responsible for historical geographical range dynamics that brought species into contact that previously were allopatric, creating opportunities for new evolutionary dynamics (Liu, Duan, et al., 2014; Liu et al., 2006; Sun et al., 2017; Xing & Ree, 2017). For all of the three hybrid speciation cases reviewed here, the hybrid species shows traits distinct from and outside the range of variation of either parental species (Lu et al., 2014; Ma et al., 2006; Sun et al., 2014). Ecological isolation may be a repeated feature of homoploid hybrid speciation in the HMR because orogenic and climatic events in this region created opportunities to develop barriers between hybrid lineages and their parents (Liu, Abbott, et al., 2014; Lu et al., 2014; Song et al., 2003; Sun et al., 2014; Wang et al., 2011, 2001). Although the importance of ecological factors in facilitating isolation between species has been recognized by several authors, in only one case for *P. densata* has ecological differentiation between the hybrid species and both parents been directly addressed through assessing distinct physiological traits for specific alpine habitats. Hence, while ecological isolation has been demonstrated, little direct evidence exists on the physiological traits associated with this divergence. More case studies directly addressing the impact of habitat dynamics on hybridization are needed (Folk et al., 2018).

According to Schumer et al. (2014), in order to have strong evidence for homoploid hybrid speciation, three criteria should be satisfied: there should be (a) reproductive barriers isolating the hybrid from its parents, (b) evidence of hybridization in the genome, and (c) demonstration that the reproductive barriers were derived directly from hybridization. Currently, only three hybrid species of *Helianthus* are known to be completely consistent with these proposed criteria (Schumer et al., 2014). However, it has been argued that these criteria have potentially artificially narrowed the importance and frequency of homoploid hybrid speciation, and by excluding ecological dimensions may fail to address factors most crucial in the generation of novel lineages (Feliner et al., 2017). Nevertheless, the role of reproductive isolation should be rigorously examined in the cases of homoploid hybrid speciation (Feliner et al., 2017).

In addition to the three homoploid hybrid species known from the HMR that are discussed above, a number of further potential cases of homoploid hybrid species in the HMR are recorded for *Rhododendron* (*R. agastum*, *R. duclouxii*), *Ligularia* (*L*. × *maoniushanensis*) and *Salix* (*S*. × *heteromera*), respectively (Pan et al., 2008; Wu et al., 2015; Zha et al., 2009; Zhang, Zhang, Gao, et al., 2007). The species described as hybrid lineages in the HMR have not formed reproductive isolation with their parental species. Additionally, to date, all homoploid hybrid species that have been documented are ecologically divergent from their parental species (Abbott & Rieseberg, 2012). These “homoploid hybrid species” from the three genus (*Rhododendron*, *Ligularia,* and *Salix*) do not ecologically or spatially divergent from its parental species. Therefore, in this review, these hybrid derivatives are considered as hybrids rather than hybrid species (see Section 4).

## 4 HYBRID ZONES IN THE HMR

Hybrid zones usually are described as regions where genetically distinct populations or species continually come into contact and mate, resulting in hybrids often of mixed ancestry (Barton & Hewitt, 1985; Harrison, 1990; Hewitt, 1988). Hybrid zones provide an opportunity as a type of “natural laboratory” for elucidating reproductive isolation mechanisms and overall the process of speciation as well as revealing dynamic patterns of introgression (Abbott et al., 2013; Harrison & Larson, 2016; Sedghifar, Brandvain, & Ralph, 2016; Taylor, Larson, & Harrison, 2015). Hybrid zones are generally a result of secondary contact triggered by migration or habitat dynamics subsequent to divergence that facilitates coexistence of divergent species (Abbott, 2017; Sousa & Hey, 2013). Reproductive isolation may constrain hybridization and formation of hybrid zones in sympatric regions, and hence often hybridization is associated with interruption of these barriers through environmental disturbance, such as natural climate change and/or human activities (Abbott, 2017). The occurrence of hybridization between divergent lineages depends on the strength of both prezygotic and postzygotic reproductive isolation (Ellstrand, Whitkus, & Rieseberg, 1996). Limited prezygotic isolation likely creates initial opportunities for interbreeding between species subsequent to divergence, while postzygotic isolation may primarily operate to maintain species distinctness despite ongoing low levels of gene flow between species.

### 4.1 The formation of hybrid zones

**Figure 2:**
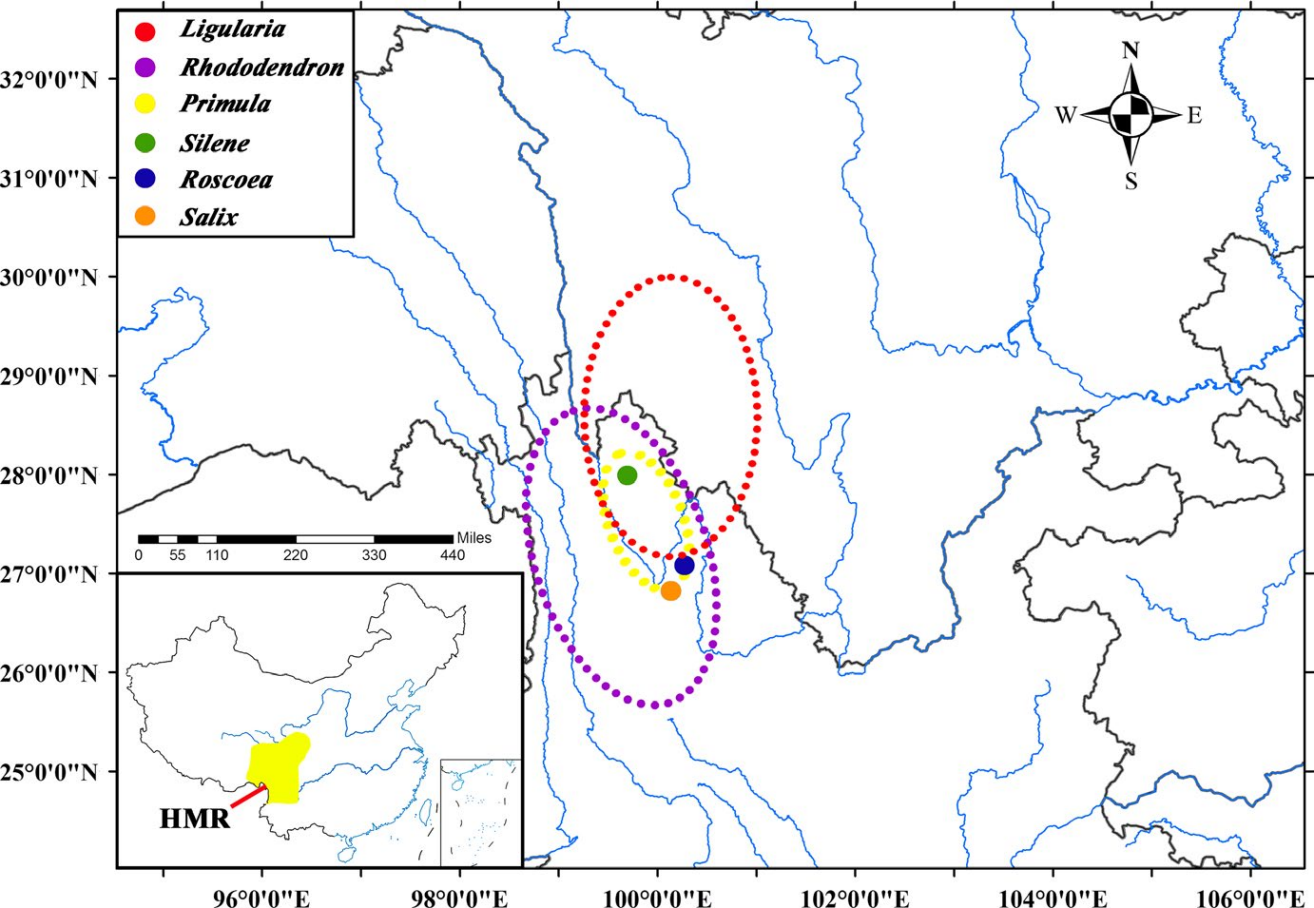
Distribution map of hybrid zones for six genera in the Hengduan mountains region. The range of the HMR is shown as yellow color area in the black rectangle (red, yellow, and purple circles represent the range of hybrid zones for *Ligularia*, *Rhododendron,* and *Primula*, respectively)

Geographical history with climate change leads to the expansion of geographical ranges that provide opportunities for secondary contact in parental species after a period of differentiation in allopatry (Abbott, 2017; Liu, Duan, et al., 2014; Sun et al., 2017). The phylogeographic studies of homoploid hybrid species in the HMR implied that the formation of ancient hybrid zones is associated with these organic events (Gao et al., 2012; Liu, Abbott, et al., 2014; Sun et al., 2014). Natural hybridization within stable hybrid zones has been reported in the HMR. Previous studies have demonstrated that several species pairs are involved, including those in *Ligularia*, *Rhododendron*, *Primula*, *Roscoea*, *Silene*, and *Salix* (Figure 2 and Table S1). Moreover, these hybrid events were potentially impacted by historic orogeny and climate as well as contemporary anthropogenic activities (Du et al., 2012; Ma, Tian, et al., 2014; Wu et al., 2015; Yang, Qin, Li, & Wang, 2012; Yu, Pan, et al., 2014; Zhang, Zhang, Gao, et al., 2007). Most of these taxa are characterized by diversification, endemism, or adaptive radiation in the HMR (Arnold & Richards, 1998; Cowley, 2007; Liu et al., 2006; Liu, Deng, & Liu, 1994; Milne, 2004; Wedderburn & Richards, 1992; Wu & Chuang, 1980). For recently diverged species, divergence time may not be enough to create efficient isolation barriers. Compared with the three homoploid hybrid species, investigation of the instances from these six taxa suggest that gene flow between the parental species mainly arose, possibly as a result of contact and human disturbance, and then generate a number of hybrids with intermediate morphological traits within hybrid zones.

A collection of species traits are prerequisites for the formation of hybrid zones, such as partially sympatric distribution together with incomplete reproductive barriers, partly overlapping flowering phenology and shared pollinator species. The six taxa, as described above, are characterized by overlapping flowering periods (Table S1). Pollinating insects in the alpine area may play a vital role in the evolution of reproductive isolation and/or hybridization for these taxa (Nie et al., 2005). In two *Rhododendron* pairs, bumblebees and honey bees are found as common pollinators (*R. delavayi* × *R. cyanocarpum* and *R. delavayi* × *R. decorum*), respectively (Ma, Zhang, Zhang, & Yang, 2010; Zhang, Feng, & Lu, 1997; Zhang, Zhang, Wu, & Qiao, 2007). Similarly, shared pollinators are observed in sympatric *Primula* (*P. secundiflora* × *P. poissonii*), *Roscoe* (*R. humeana* × *R. cautleoides*) (Du et al., 2012; Zhu et al., 2009). Generalist pollination syndromes are thought in particular to be a key feature for hybridization in *Ligularia* (Cao, Ma, & Wang, 2008; Yu, Kuroda, & Gong, 2011; Yu, Kuroda, et al., 2014). Therefore, these prerequisites may reduce reproductive barriers and increase the probability for occurrence of hybridization in regions of sympatry for these taxa.

### 4.2 The effect of reproductive barriers on hybrid zones

Reproductive isolating mechanisms can be categorized into two principle types: prezygotic and postzygotic (Baack, Melo, Rieseberg, & Ortiz‐Barrientos, 2015; Rieseberg & Carney, 1998; Rieseberg & Willis, 2007). Prezygotic isolation in plants comprises habitat, temporal, ethological, and gametic competition or incompatibility, while postzygotic isolations are mainly comprised of hybrid weakness or inviability, hybrid breakdown (Rieseberg & Carney, 1998). Because postzygotic isolation is costly due to gamete wastage, prezygotic isolation has been considered to be more important than postzygotic isolation for sympatric species (Baack et al., 2015). The dynamic and architecture of hybrid zones are potentially affected by both isolation mechanisms (Rieseberg & Carney, 1998).

Asymmetrical hybridization is a relative common phenomenon in the plant kingdom (Arnold, 1997; Barton & Hewitt, 1985). Multiple reproductive isolation mechanisms may potentially affect the direction of hybridization and introgression (Arnold, Tang, Knapp, & Martin, 2010). Bidirectional and asymmetrical hybridization typically are both commonly observed in hybrid zones of the HMR, although two instances reveal the sole occurrence of unidirectional hybridization in *Primula* and *Salix* (Ma, Tian, et al., 2014; Wu et al., 2015). Asymmetrical hybridization may be largely attributed to both types of barriers in hybrid zones of the HMR. Both pre‐ and postzygotic isolation mechanisms usually result in a tendency toward asymmetrical hybridization that leads to favoring one of the species as the maternal species (Ma, Xie, Sun, & Marczewski, 2016). Recently diverged species usually have unequal strength in isolation barriers and/or in reproductive output, potentially leading to asymmetrical gene flow in sympatric zones. Examples include differences in phenology, pollinator preference, genetic incompatibility strength, and local abundance of parents (Carney, Gardner, & Rieseberg, 2000; Muranishi, Tamaki, Setsuko, & Tomaru, 2013; Zhou, Gong, Boufford, Wu, & Shi, 2008).

First, at least for protandrous species, the species that flowers earlier than another would be more likely maternal species, while pollen from the later flowering parent is more likely to be received. Flowering phenology has been shown to be a crucial prezygotic isolation in the HMR (Table S1).

Protandry, a key feature for *Rhododendron*, combined with flowering time may lead to generate hybrid asymmetry such that gene flow is strongly biased toward favoring an earlier flowering species as the maternal parent in this genus (Ma, Milne, et al., 2010; Milne & Abbott, 2008; Zha, Milne, & Sun, 2008, 2009). Among three species pairs in this genus, maternally inherited plastid DNA (cpDNA) markers (Harris & Ingram, 1991; Olmstead & Palmer, 1994) have shown that most hybrid offspring share chloroplast haplotypes with *R. delavayi*, which previous studies have shown flowers earlier than *R. cyanocarpum*, *R. decorum,* and *R. irroratum* (Ma, Milne, et al., 2010; Zha, Milne, & Sun, 2008, 2009; Zhang, Zhang, Gao, et al., 2007; Zheng et al., 2017). Hence, it has been inferred that hybridization in this system is strongly biased toward *R. delavayi* as the maternal parent. With another similar case from *Silene*, hybrid individuals shared more cpDNA haplotypes with *S. asclepiadea* than with *S. yunnanensis*, suggesting *S. asclepiadea* is main maternal species. Similarly, protandry has been found in the both parental species and the peak flowering of *S. yunnanensis* is later than *S. asclepiadea* about 10 days. Therefore, temporal asynchronism of the flowering phenology has been thought contributing to asymmetric hybridization in this species pair (Zhang et al., 2016). Likewise, the effect of flowering time without protandry on hybridization has been presented in other taxa. *Ligularia subspicata* flowers slightly earlier than *L. nelumbifolia*. Accordingly, on the basis of cpDNA markers, *L. subspicata* appears to have been primarily the maternal parent (Yu et al., 2011).

Secondly, asymmetrical hybridization can potentially arise from differences in pollen quantity. In the case of “pollen swamping,” it is predicted that the rare species may generally act as the maternal parent relative to more abundant species (Arnold, Hamrick, & Bennett, 1993; Lepais et al., 2009; Levin, Francisco‐Ortega, & Jansen, 1996; Rieseberg, 1995). Gene flow patterns may competitively favor individuals or flowers either with greater pollen production or that are greater in frequency in the hybrid zone. Moreover, difference in competition of the pollen at the stigmatic surface may facilitate asymmetrical hybridization. The bidirectional and asymmetrical hybridization are suggested in the case of *L. subspicata* and *L. nelumbifolia* (Yu et al., 2011). One of the parents is five times more prevalent than the other in a sympatric zone of *Ligularia*. In addition, compound corymb inflorescences can produce more pollen in some *Ligularia*, such as *L. nelumbifolia* (more than 100 cephaloid with 6–8 florets). In addition, the number of *L. duciformis* is more than *L. yunnanensis*. Likewise, in *L. duciformis* and *L. yunnanensis*, the relative abundance of parental species may result to bias in *L. yunnanensis* as maternal parent although both species have similar floral traits (Zhang et al., 2018). Hence, a number of factors may cause asymmetrical hybridization in the *Ligularia* species pair (Yu et al., 2011).

Finally, pollinator‐mediated (ethological) isolation may be another key prezygotic barrier in plants (Table S1). In the species pair *P. secundiflora* and *P. poissonii*, pollinator syndromes may contribute to an asymmetrical reproductive barrier in *Primula* (Xie et al., 2017). Hymenoptera are the main floral visitors of *P. secundiflora* (e.g., bumblebees and *Anthophora*), while ca 30% of pollinators are from Lepidoptera in *P. poissonii* (Xie et al., 2017). Thus, prezygotic pollinator‐mediated isolation is thought to be an important factor for asymmetrical gene flow in the *Primula* species pair (Xie et al., 2017). The same reasons may also result in asymmetrical hybridization in *Silene*. Butterflies are the main visitors to *S. yunnanensis*, while bumblebees trend to *S. asclepiadea* and hybrid individuals (Zhang et al., 2016). In the case of *Rhododendron*, no evidence of gene flow bias has been found in *R. spiciferum* and *R. spinuliferum*, yet these differ in pollinators, where bird and bee pollination are present in *R. spinuliferum* and *R. spiciferum*, respectively (Yan et al., 2013). Larger compound corymb inflorescences in *L. duciformis* favor this species as the maternal parent in hybridizing with the racemose species *L. cyathiceps* because the floral traits of former are more attractive to pollinators (Zhang et al., 2018). Consequently, pollinator‐mediated may lead to asymmetrical barriers and/or hybridization in these taxa of the HMR.

Postzygotic isolation may also contribute to maintain hybrid zones in the HMR. The weakness of many prezygotic isolation mechanisms may often provide opportunities for interspecific incomplete reproductive isolation and gene flow in the six taxa as described above (Table S1). However, postzygotic isolation can prevent genetic homogenization of both species, which is one potential outcome from hybridization and introgression (Rieseberg & Carney, 1998). Because these types of barriers are difficult to lose once evolved, postzygotic isolation may also be an important mechanism influencing the dynamics and structure of hybrid zones (Coyne & Orr, 2004; Orr, 1996; Orr & Turelli, 2001). Postzygotic isolation may also limit interspecific hybridization or the formation of novel hybrid lineage when prezygotic isolation was permeable.

For *P. secundiflora* and *P. poissonii*, postzygotic barriers restrain hybridization via selection against F_1_ hybrids (Table S1), which show much lower seed numbers, high rate of seed inviability, embryo developmental failure, and low germination rates. The differences between the two parental lineages in heteromorphic incompatibility of pollen potentially also contributes to the asymmetrical strength of reproductive isolations in the hybridizing species pair (Xie et al., 2017). The predominance of the F_1_ hybrid generation is commonly reported in hybrid zones of the HMR, a structure that likely arises from pre‐existing strong postzygotic isolation among parental species (Table S1). In two hybrid zones of *Rhododendron*, all hybrid individuals are categorized as F_1_ in one zone (HuaDianBa), while most individuals of F_1_ with small numbers of other classes are dominated in another zone (ZhuJianYuan) (Zha et al., 2009). Because selection potentially acts against later‐generation hybrid derivatives, habitat mediation may result in the difference between both the hybrid zones in hybrid fitness (Zha et al., 2009). In three further HMR species pairs, the F_1_ generation is primary hybrid component (Du et al., 2012; Yu et al., 2011; Zhang et al., 2018). In the case of *L. subspicata* and *L. nelumbifolia*, extremely low seed germination rate of hybrid offspring (3‰) suggests the presence of strong postzygotic barriers may occur in this species pair (Yu et al., 2011). Likewise, in the three sympatric *Ligularia* case, hybridization only occurred in *L. cyathiceps* and *L. duciformis* or *L. duciformis* and *L. yunnanensis*. However, hybrids between *L. cyathiceps* and *L. yunnanensis* have not been detected (Zhang et al., 2018). Genetic data indicate relatively higher genetic distance between *L. cyathiceps* and *L. yunnanensis* than either has with *L. duciformis*. Hence, mutations between the both species may have been accumulated to build postzygotic isolation by sterility of hybrids (Zhang et al., 2018). In the case of *R. humeana* and *R. cautleoides*, F_1_ hybrid individuals are found in the wild (Du et al., 2012). Unlike hybrid species distributed in novel or extreme habitats, only isolated, presumably ephemeral hybrid individuals have been found in intermediate habitats, suggesting these hybrids are not stabilized. However, in other cases from *Ligularia* and *Rhododendron*, F_2_ hybrids are the dominated class in hybrid zones (Ma, Milne, et al., 2010; Zhang et al., 2017). Overall, these cases suggest that incomplete postzygotic barriers may still have impact on the fitness of hybrid offspring, and this process may be responsible for the maintenance of species boundaries despite frequent hybridization in the HMR.

Sufficient fertile F_1_ hybrids can produce backcrosses and act as potential genetic bridge between both parental species for introgression (Cannon & Scher, 2017; Harrison et al., 2017; Twyford, Kidner, & Ennos, 2015; Yatabe, Kane, Scotti‐Saintagne, & Rieseberg, 2007). Hence, frequent backcrossing may lead to introgression of genetic material to one or both parents. As discussed above, asymmetrical hybridization is a commonly observed phenomenon in the HMR. Thus, distinct patterns of isolation between species likely generate asymmetrical introgression in many of the hybrid zones of the HMR (Table S1). Flower traits of hybrids may have similar morphology with its parental species due to repeated backcrosses occurring in hybrid zone (Abbott et al., 2013; Arnold, 1997). In a species pair of *Primula*, asymmetrical hybridization (unidirectional admixture with maternal *P. bulleyana*) and repeated backcrossing may have resulted in more hybrids having phenotypes similar to *P. bulleyana*, and genetic composition may be introgressed from *P. beesiana* into *P. bulleyana* (Ma, Tian, et al., 2014; Ma, Xie, et al., 2014). Open and shaded habitats are occupied by *P. beesiana* and *P. bulleyana*, respectively (Ma, Tian, et al., 2014), whereas *P. bulleyana* ‐like hybrids individuals have been found in open habitat. Therefore, introgression may transferred novel phenotypes from *P. beesiana* to *P. bulleyana* (Ma, Tian, et al., 2014; Ma, Xie, et al., 2014); this introgression may have been and enabled the colonization of new habitats (Arnold, 1997; Arnold, Ballerini, & Brothers, 2012; Zulliger, Schnyder, & Gugerli, 2013). Although postzygotic barriers remain uncharacterized in other hybrid zones of the HMR, to date the postzygotic mechanisms above may be generally responsible for the frequent occurrence of asymmetrical introgression (Ma, Milne, et al., 2010; Xie et al., 2017; Yu et al., 2011; Yu, Pan, et al., 2014; Zha et al., 2009; Zhang et al., 2016). More work is required to determine how reproductive barrier affect the structure of hybrid zones and reflect selection. Species boundaries have been thought to be “semipermeable” (Harrison & Larson, 2014).

A combination of genetic and ecological analyses should be implemented to explore these issues, especially for intrinsic and extrinsic barriers to hybridization. Genomic analyses will help to unravel how gene flow between divergent lineages can produce novel morphological diversity for adaption to new habitats. Investigation of molecular and biochemical mechanisms in hybrid incompatibility could improve the understanding of the relationship between reproductive isolation and environment (Chen, E, & Lin, 2016).

## 5 CONCLUSIONS

Interspecific hybridization may be among the factors responsible for higher plant diversity in montane regions (Liu, Duan, et al., 2014; Liu et al., 2006; Nie et al., 2005; Sun et al., 2017; Wen et al., 2014; Xing & Ree, 2017). According to our review, approximately 18 species pairs from nine genera are suggested to be involved in homoploid hybridization. Based on the evidence accumulated to date, homoploid hybridization appears to be a general phenomenon in the flora of the HMR.

The majority of species pairs are believed to form hybrid zones arising from incomplete isolation (reproductive and/or ecological barriers). Incomplete reproductive isolation is likely important for maintaining hybrid zones in these montane regions, since it can lead to asymmetrical hybridization, introgression, and species boundaries maintenance (Rieseberg & Blackman, 2010). In addition to naturally occurring ecological dynamics, a substantial number of hybrid zones known from the HMR appear in the context of anthropogenic disturbance, often in roadside situations. Unfortunately, there have been few detailed how the distribution of human activities has affected hybridization of the HMR. Hence, the effect of anthropogenic disturbance on natural hybridization, that is, reproductive barriers, should be taken into account.

The ecological niche of hybrids could diverge from the niche space occupied by parental species if hybrid zones generate hybrid lineages with novel trait combinations (Rieseberg et al., 2007). Three homoploid hybrid speciation events indicate that ecological isolations resulting from topological diversity may greatly facilitate the formation of reproductive barriers between parental species and hybrid species. Therefore, complex and dynamic geographical and climatic conditions, characteristic of the HMR, may create ecological opportunities that trigger hybridization and/or speciation (Liu, Duan, et al., 2014; Liu et al., 2006; Wen et al., 2014; Xing & Ree, 2017). Ecological divergence may be the most likely force to promote stabilization of hybrids and create opportunities for homoploid hybrid speciation in the HMR. Therefore, hybrid zones may be a key step for homoploid hybrid speciation.

The high occurrence in this mountains region possibly implies that plant hybrid zones may be partly responsible for genetic variation involved in the evolution and diversification of flora in the HMR. However, in these case studies, extrinsic and intrinsic reproductive barriers are still insufficiently known at the molecular and ecological levels. Hence, future efforts in understanding hybridization in the HMR should focus on dissecting ecological and molecular mechanism of reproductive isolation that may be responsible for these observed patterns. Future studies should focus on the mechanisms of reproductive isolation and ecological niche shift in these alpine hybrid zones. Understanding of these mechanisms will help evolutionary biologist to identify the role of homoploid hybridization in adaptive radiation of plants in the hyper‐diverse HMR.

## CONFLICT OF INTEREST

None declared.

## AUTHOR CONTRIBUTIONS

XG contributed conception of the manuscript; RY wrote the manuscript; RF provided substantial feedback on the manuscript and approved the English language for the manuscript; NN‐Z reviewed the paper; all authors contributed to manuscript revision, read and approved the submitted version.

## Data Availability

No new data was created in the course of this research.
