# Supplementary material for: Homoploid hybridization of plants in the Hengduan mountains region
Source: Ecol Evol. 2019 Jun 23;9(14):8399–410. doi: 10.1002/ece3.5393 (PMC6662326; doi:10.1002/ece3.5393)
Supplement: Supplementary file 1 [file ECE3-9-8399-s001.docx]

**TABLE S1** Species pair for taxa, hybrid direction, introgression, hybrids composition, isolation barriers, habitat disturbance, genetic markers and main conclusion for 18 pairs of nine genus

| Genus | Taxa | Hybrid direction | Introgression | Hybrids composition | Isolating barriers | Habitat disturbance | Genetic markers | References |
| --- | --- | --- | --- | --- | --- | --- | --- | --- |
| *Pinus* | *P. yunnanensis* × *P. tabuliformis* | Bidirectional and asymmetrical hybridization appear in ancient the initial stage of hybridization and speciation. The historical gene flow mainly towards *P. tabuliformis*. | Distinct extent in the different ancient hybrid zones. | Possible backcross individuals appear in *P. yunnanensis* populations in partial overlap region. | Ecological niche differentiate from parental species with tectonic activities and climatic change. | Tectonic change and climatic oscillation in history. | cpDNA, RFLP, mtDNA, EST | Gao et al., 2012; Ma et al., 2010; Ma, Szmidt, & Wang, 2006; Song, Wang, Wang, Ding, & Hong, 2003; Song et al., 2002; Wang, Mao, Gao, Zhao, & Wang, 2011; Wang & Szmidt, 1990; Wang, Szmidt, & Savolainen, 2001 |
| *Picea* | *P. wilsonii* × *P. purpurea* | Bidirectional hybridization occur in overlap region between *P. wilsonii* and *P. purpurea*. | Based on mtDNA, *P. purpurea* is introgressed by genetic background from *P. likiangensis*, while bidirectional introgression is detected in cpDNA. | According to both cpDNA and mtDNA, backcross individuals may occur in overlap region between *P. wilsonii* and *P. purpurea*. | Hybrid species *P. purpurea* have unique genetic materials Although hybrid species share similar alleles with both parental species. *P. purpurea* occupied ecological niche which is differentiated from parents. | Historical climatic change. | mtDNA, cpDNA | Du et al., 2011; Sun et al., 2014 |
| *Ostryopsis* | *O. davidiana* × *O. nobilis* | The estimate of gene flow only occurred in historical sympatric region. | Genetic materials of *O. davidiana* are transferred into *O. nobilis* based on cpDNA. *O. davidiana* haplotypes are possibly introgressed to early diverged *O. nobilis*. | No stated. | Hybrid species *O. intermedia* may expand its distribution to new ecological niche with founder effect. | Glaciation period and geographic change. | ITS, cpDNA, AFLP, nDNA | Liu, Abbott, Lu, Tian, & Liu, 2014; Lu, Tian, Liu, Yang, & Liu, 2014; Tian, Liu, & Liu, 2010 |
| *Ligularia* | *L. paradoxa* × *L. duciformis* | According to cpDNA, one of both hybrid zones (Mt. Maoniu) suggest unidirectional hybridization is appeared with *L. paradoxa* as maternal species, while bidirectional and asymmetrical hybridization is occurred in another zone with *L. duciformis* as predominant maternal species (Heihai Lake). | Some individuals of *L. duciformis* or *L. paradoxa* are detected as introgressive individuals. Not stated for introgressive direction. | F_2_ hybrids and backcross individuals are generally appeared in both hybrid zones. | Strong post-zygotic barrier possibly occur between both species due to none of viable seeds are found in hybrid species *L. ×maoniushanensis*. | Not stated. | ISSR, cpDNA, SSR, ITS | Pan, Shi, Gong, & Kuroda, 2008; Zhang, Gong, & Ryan, 2017 |
|  | *L. nelumbifolia* × *L. subspicata* | Gene flow are revealed in bidirectional and asymmetrical hybridization with *L. subspicata* is inferred as primary maternal species. | Not stated. | F_1_ are more prevalent than F_2_ or post-F_2_. | Slightly distinct flowering are appeared in sympatric zone, suggesting possible pre-zygotic barrier are occurred between both species. Restricted post-zygotic isolation may appear between parental species. | Yes: meadow after fire disturbance. | cpDNA, ISSR, ITS | Yu, Kuroda, & Gong, 2011 |
|  | *L. vellerea* × *L. subspicata* | Maternal species are detected in all of *L. subspicata*, based on cpDNA, for unidirectional hybridization. However, bidirectional hybridization are detected in later research. | Not stated. Introgression may occur in the hybrid zone. | F_2_ or post-F_2_ and backcrosses are possibly occur in the sympatric zone. | The anthesis of *L. vellerea* is slight earlier than *L. subspicata*, suggesting incomplete pre-zygotic barrier may occur between parents. Post-zygotic isolation may appear in the hybrid zone. | Yes: road building. | ITS, cpDNA | Ning, Yu, & Gong, 2017; Yu, Pan, Pan, & Gong, 2014 |
|  | *L. cymbulifera* × *L. tongolensis* | Different hybrid direction are found among four hybrid zones. | Except for Desha hybrid zone, three mixed sites have introgressed individuals of *L. tongolensis*, whereas introgressed individuals of *L. cymbulifera* are only found in Desha. Not stated for introgressive direction. | F_1_ or post-F_1_ may occur in four hybrid zones, because introgressed individuals are detected in these locations. | Only partial overlap of anthesis are present in sympatric regions, suggesting a certain degree of pre-zygotic barrier may occur between both species. Pure parental species are found in hybrid zone, implying post-zygotic barrier may contribute to maintain species boundaries. | Not stated. Four hybrid zones occur in the side of road, thus these hybrid zones may be affected by road building. | ITS, cpDNA | Yu, Kuroda, & Gong, 2014 |
|  | *L. cyathiceps* × *L. duciformis*;  *L. duciformis* × *L. yunnanensis* | Bidirectional but asymmetric hybridization appear in both groups.  *L. cyathiceps* × *L. duciformis*: *L. duciformis* is the primary maternal parent;  *L. duciformis* × *L. yunnanensis*: *L. yunnanensis* is the maternal parent | Not stated. | F_1_ dominated in both hybridizing groups. | Pre-zygotic: incomplete barriers.  Post-zygotic: the accumulation of mutations between *L. cyathiceps* and *L. yunnanensis*. | Yes: human activities such as tree felling and grazing | cpDNA, nDNA | Zhang, Yu, Wang, & Gong, 2018 |
| *Rhododendron* | *R. cyanocarpum* × *R. delavayi* | Bidirectional and asymmetrical hybridization occur in the hybrid zone with *R. delavayi* as major maternal species. | Introgression may appear in the hybrid zone. | The hybrid zone are comprised by hybrid swarm, i.e. F_1_, F_2_ and backcrossing individuals of *R. delavayi*, which is dominated by F_2_. | Pre-zygotic: Geographic and phenological distinction are main barriers in *R. delavayi*. Pollinator constancy play an important role in reproductive barrier between both species. | Yes: habitat disturbance from deforestation. | cpDNA, nDNA, ITS, AFLP | Ma, Milne, Zhang, & Yang, 2010; Ma, Xie, Sun, & Marczewski, 2016; Ma, Zhang, Zhang, & Yang, 2010 |
|  | *R. delavayi* × *R. decorum* | Bidirectional and asymmetrical hybridization with *R. delavayi* as major maternal species. | Not stated. | F_1_, F_2_ and backcrossed individuals. | The difference in flowering times between both parental species probably contribute to pre-zygotic isolation. Post-zygotic barrier occur between species. | Yes: habitat disturbance of deforestation and the development of tourism. | cpDNA, ITS, AFLP | Zha, Milne, & Sun, 2008; Zhang, Zhang, Gao, Yang, & Li, 2007; Zheng et al., 2017 |
|  | *R. irroratum* × *R. delavayi* | Two hybrid zones (HDB and ZJY): HDB, unidirectional hybridization with *R. delavayi* as maternal species; ZJY, bidirectional and asymmetrical hybridization with *R. delavayi* mainly play a role of maternal species. | Only ZJY site may have introgression, but not further stated. | F_1_ dominate HDB site, while ZJY is comprised by F_1_ and fewer post-F_1_ or F_2_. | Unilateral incompatibility, phenology and pollinator behaviour may contribute pre-zygotic isolation in both hybrid zones. Strong post-zygotic isolation may affect the fitness of hybrids. | Yes: habitat disturbance of deforestation. | cpDNA, ITS, AFLP | Zha, Milne, & Sun, 2009 |
|  | *R. aganniphum* × *R. phaeochrysum* | Two hybrid zones (hyb-1 and hyb-2): hyb-1, unidirectional hybridization with gene flow from *R. phaeochrysum* to *R. aganniphum*; hyb-2, the absence of backcrossed individuals prevent gene flow. | Large proportion of loci are suggested to be affected by introgression. Not clearly for introgressive direction. | Hyb-1: F_1_ and backcrossed individuals of *R. aganniphum*; hyb-2: only F_1_. | Incompatibilities most likely to affect F_1_ hybrids. | No. | AFLP | Marczewski, Chamberlain, & Milne, 2015 |
| *Primula* | *P. secundiflora* × *P. poissonii* | Bidirectional or bidirectional but asymmetrical hybridization with *P. poissonii* as major maternal species. | Introgression may occur in one of two hybrid zones (Potatso national park site). | Only Potatso national park site identified the composition of hybrids: F_1_ and backcrossed individuals of *P. poissonii*. | Pre-zygotic: the differentiation of pollinator mainly contribute pre-zygotic reproductive isolation; Different degree of heteromorphic incompatibility between species; Heterostyly; Strong post-zygotic isolation maintain species boundaries. | Yes: Grazing activity. | cpDNA, ITS, SSR | Xie et al., 2017; Xie, Zhao, Zhu, Li, & Li, 2017; Zhu et al., 2009 |
|  | *P. beesiana* × *P. bulleyana* | Unidirectional hybridization with *P. bulleyana* as maternal species. | Adaptive introgression had been detected in the hybrid zone. Genetic materials of *P. beesiana* are mainly transferred to *P. bulleyana*. | Rare F_1_ and possible post-F_1_. | Incomplete pre-zygotic isolation: Partial ecological isolation; heterospecific pollen incompatibility; Post-zygotic: lower reproductive success for synthetic F_1_s. | Yes: Road building for tourism development. | cpDNA, ITS, AFLP | Ma, Tian, Zhang, Wu, & Sun, 2014; Ma et al., 2014 |
| *Roscoea* | *R. humeana* × *R. cautleoides* | Possible bidirectional hybridization with *R. humeana* as main maternal species. | Not clearly. | F_1_ dominated in hybrid zone. | Incomplete pre-zygotic and post-zygotic isolations. | Not clearly. | HAT-RAPD | Du, Hui., & Jun., 2011; Du, Zhang, & Li, 2012 |
| *Salix* | *S. matsudana* × *S. cavaleriei* | Unidirectional hybridization with *S. cavaleriei* as maternal species. | Not stated. | Not stated. Hybrids (*S.* ×*heteromera*) only occur in both parental sympatric zone. | Not stated. Post-zygotic barriers may occur between both parental species: the restricted distribution of hybrids are associated with the infertility or inviability of hybrids or its offspring. | Yes: invasion species (*S. matsudana*) are introduced by human for ornamental plant; High frequency of anthropogenic activities. | cpDNA, ITS | Wu, Wang, Yang, & Chen, 2015 |
| *Silene* | *S. asclepiadea* × *S. yunnanensis* | Bidirectional but asymmetric hybridization with *S. asclepiadea* as main maternal species. | Not clearly. | Not stated. | Pre-zygotic: Phenology, a unilateral pre-zygotic barrier, pollinator preference and low pollen production. Post-zygotic: Low viable seed production | Not stated. | cpDNA, SSR | Zhang, Montgomery, & Huang, 2016 |

Genetic markers: cpDNA, chloroplast DNA; mtDNA, mitochondrial DNA; nDNA, nuclear DNA; HAT-RAPD, high annealing temperature-randomly amplified polymorphic DNA; nrETS, the external transcribed spacer of nuclear ribosomal DNA; AFLP, amplified fragment length polymorphisms; SSR, simple single repeats; ITS, nuclear ribosomal internal transcribed spacer sequence; RFLP, restriction fragment length polymorphism; nSSR, nuclear simple sequence repeat; ISSR, inter simple sequence repeat.

**REFERENCES FOR TABLE S1**

Du, F. K., Peng, X. L., Liu, J. Q., Lascoux, M., Hu, F. S., & Petit, R. J. (2011). Direction and extent of organelle DNA introgression between two spruce species in the Qinghai-Tibetan Plateau. *New Phytologist, 192*(4), 1024-1033. doi:http://dx.doi.org/10.1111/j.1469-8137.2011.03853.x

Du, G., Hui., L., & Jun., Q. (2011). Application of HAT-RAPD technique in identifying natural hybrids of *Roscoea* (Zingiberaceae). *Plant Diversity and Resources, 33*(6), 683-689. doi:10.1631/jzus.B1000171

Du, G. H., Zhang, Z. Q., & Li, Q. J. (2012). Morphological and molecular evidence for natural hybridization in sympatric population of *Roscoea humeana* and *R. cautleoides* (Zingiberaceae). *Journal of Plant Research, 125*(5), 595-603. doi:http://dx.doi.org/10.1007/s10265-012-0478-6

Gao, J. I. E., Wang, B., MAO, J. F., Ingvarsson, P., ZENG, Q. Y., & WANG, X. R. (2012). Demography and speciation history of the homoploid hybrid pine *Pinus densata* on the Tibetan Plateau. *Molecular Ecology, 21*(19), 4811-4827. doi:https://doi.org/10.1111/j.1365-294X.2012.05712.x

Liu, B., Abbott, R. J., Lu, Z., Tian, B., & Liu, J. (2014). Diploid hybrid origin of *Ostryopsis intermedia* (Betulaceae) in the Qinghai-Tibet Plateau triggered by Quaternary climate change. *Molecular Ecology, 23*(12), 3013-3027. doi:https://doi.org/10.1111/mec.12783

Lu, Z. Q., Tian, B., Liu, B. B., Yang, C., & Liu, J. Q. (2014). Origin of *Ostryopsis intermedia* (Betulaceae) in the southeast Qinghai-Tibet Plateau through hybrid speciation. *Journal of Systematics and Evolution, 52*(3), 250-259. doi:https://doi.org/10.1111/jse.12091

Ma, F., Zhao, C., Milne, R., Ji, M., Chen, L., & Liu, J. (2010). Enhanced drought-tolerance in the homoploid hybrid species *Pinus densata*: implication for its habitat divergence from two progenitors. *New Phytologist, 185*(1), 204-216. doi:http://dx.doi.org/10.1111/j.1469-8137.2009.03037.x

Ma, X. F., Szmidt, A. E., & Wang, X. R. (2006). Genetic structure and evolutionary history of a diploid hybrid pine *Pinus densata* inferred from the nucleotide variation at seven gene loci. *Molecular Biology and Evolution, 23*(4), 807-816. doi:http://dx.doi.org/10.1093/molbev/msj100

Ma, Y. P., Milne, R. I., Zhang, C., & Yang, J. (2010). Unusual patterns of hybridization involving a narrow endemic *Rhododendron species* (Ericaceae) in Yunnan, China. *American Journal of Botany, 97*(10), 1749-1757. doi:http://dx.doi.org/10.3732/ajb.1000018

Ma, Y. P., Tian, X. L., Zhang, J. L., Wu, Z. K., & Sun, W. B. (2014). Evidence for natural hybridization between *Primula beesiana* and *P. bulleyana*, two heterostylous primroses in NW Yunnan, China. *Journal of Systematics and Evolution, 52*(4), 500-507. doi:https://doi.org/10.1111/jse.12077

Ma, Y. P., Xie, W., Tian, X., Sun, W. B., Wu, Z. K., & Milne, R. (2014). Unidirectional hybridization and reproductive barriers between two heterostylous primrose species in north-west Yunnan, China. *Annals of Botany, 113*(5), 763-775. doi:http://dx.doi.org/10.1093/aob/mct312

Ma, Y. P., Xie, W. J., Sun, W. B., & Marczewski, T. (2016). Strong reproductive isolation despite occasional hybridization between a widely distributed and a narrow endemic *Rhododendron* species. *Scientific Reports, 6*, 19146. doi:http://dx.doi.org/10.1038/srep19146

Ma, Y. P., Zhang, C. Q., Zhang, J. L., & Yang, J. B. (2010). Natural hybridization between *Rhododendron delavayi* and *R. cyanocarpum* (Ericaceae), from morphological, molecular and reproductive evidence. *Journal of Integrative Plant Biology, 52*(9), 844-851. doi:http://dx.doi.org/10.1111/j.1744-7909.2010.00970.x

Marczewski, T., Chamberlain, D. F., & Milne, R. I. (2015). Hybridization in closely related *Rhododendron* species: half of all species-differentiating markers experience serious transmission ratio distortion. *Ecology and evolution, 5*(15), 3003-3022. doi:https://doi.org/10.1002/ece3.1570

Ning, H., Yu, J., & Gong, X. (2017). Bidirectional natural hybridization between sympatric *Ligularia vellerea* and *L. subspicata*. *Plant Diversity, 39*(4), 214-220. doi:10.1016/j.pld.2017.07.001

Pan, Y., Shi, S., Gong, X., & Kuroda, C. (2008). A Natural Hybrid Between *Ligularia paradoxa* and *L. duciformis* (Asteraceae, Senecioneae) From Yunnan, China. *Annals of the Missouri Botanical Garden, 95*(3), 487-494. doi:https://doi.org/10.3417/2006034

Song, B. H., Wang, X. Q., Wang, X. R., Ding, K. Y., & Hong, D. Y. (2003). Cytoplasmic composition in *Pinus densata* and population establishment of the diploid hybrid pine. *Molecular Ecology, 12*(11), 2995-3001. doi:http://dx.doi.org/10.1046/j.1365-294X.2003.01962.x

Song, B. H., Wang, X. Q., Wang, X. R., Sun, L. J., Hong, D. Y., & Peng, P. H. (2002). Maternal lineages of *Pinus densata*, a diploid hybrid. *Molecular Ecology, 11*(6), 1057-1063. doi:http://dx.doi.org/10.1046/j.1365-294X.2002.01502.x

Sun, Y., Abbott, R. J., Li, L., Li, L., Zou, J., & Liu, J. (2014). Evolutionary history of purple cone spruce (*Picea purpurea*) in the Qinghai-Tibet Plateau: homoploid hybrid origin and Pleistocene expansion. *Molecular Ecology, 23*(2), 343-359. doi:http://dx.doi.org/10.1111/mec.12599

Tian, B., Liu, T. L., & Liu, J. Q. (2010). *Ostryopsis intermedia*, a new species of Betulaceae from Yunnan, China. *Botanical Studies, 51*(2), 257-262. doi:10.1515/BOT.2010.022

Wang, B., Mao, J. F., Gao, J. I. E., Zhao, W. E. I., & Wang, X. R. (2011). Colonization of the Tibetan Plateau by the homoploid hybrid pine *Pinus densata*. *Molecular Ecology, 20*(18), 3796-3811. doi:http://dx.doi.org/10.1111/j.1365-294X.2011.05157.x

Wang, X. R., & Szmidt, A. E. (1990). Evolutionary analysis of *Pinus densata* (Masters), a putative Tertiary hybrid. *Theoretical and Applied Genetics, 80*(5), 641-647. doi:http://dx.doi.org/10.1007/BF00224224

Wang, X. R., Szmidt, A. E., & Savolainen, O. (2001). Genetic composition and diploid hybrid speciation of a high mountain pine, *Pinus densata*, native to the Tibetan plateau. *Genetics, 159*(1), 337-346. doi:10.1089/10906570152742353

Wu, J., Wang, D. C., Yang, Y. P., & Chen, J. H. (2015). Homoploid hybridization between native *Salix cavaleriei* and Exotic *Salix matsudana* (Salicaceae). *Plant Diversity and Resources, 37*(1), 1-10. doi:10.7677/ynzwyj201514052

Xie, Y., Zhu, X., Ma, Y., Zhao, J., Li, L., & Li, Q. (2017). Natural hybridization and reproductive isolation between two *Primula* species. *Journal of Integrative Plant Biology, 59*, 526-530. doi:http://dx.doi.org/10.1111/jipb.12546

Xie, Y. P., Zhao, J. L., Zhu, X. F., Li, L., & Li, Q. J. (2017). Asymmetric hybridization of *Primula secundiflora* and *P. poissonii* in three sympatric populations. *Biodiversity Science, 25*(6), 647-653. doi:10.17520/biods.2017001

Yu, J. J., Kuroda, C., & Gong, X. (2011). Natural hybridization and introgression in sympatric *Ligularia* species (Asteraceae, Senecioneae). *Journal of Systematics and Evolution, 49*(5), 438-448. doi:http://dx.doi.org/10.1111/j.1759-6831.2011.00150.x

Yu, J. J., Kuroda, C., & Gong, X. (2014). Natural hybridization and introgression between *Ligularia cymbulifera* and *L. tongolensis* (Asteraceae, Senecioneae) in four different locations. *PloS One, 9*(12), e115167. doi:http://dx.doi.org/10.1371/journal.pone.0115167

Yu, J. J., Pan, L., Pan, Y. Z., & Gong, X. (2014). Natural hybrids between *Ligularia vellerea* and *L. subspicata* (Asteraceae: Senecioneae). *Plant biodiversity and resources, 36*, 219-226. doi:10.7677/ynzwyj201413109

Zha, H. G., Milne, R. I., & Sun, H. (2008). Morphological and molecular evidence of natural hybridization between two distantly related *Rhododendron* species from the Sino-Himalaya. *Botanical Journal of the Linnean Society, 156*(1), 119-129. doi:http://dx.doi.org/10.1111/j.1095-8339.2007.00752.x

Zha, H. G., Milne, R. I., & Sun, H. (2009). Asymmetric hybridisation in *Rhododendron agastum*: a hybrid taxon comprising mainly F1s in Yunnan, China. *Annals of Botany, 105*(1), 89-100. doi:10.1093/aob/mcp267

Zhang, J. J., Montgomery, B. R., & Huang, S. Q. (2016). Evidence for asymmetrical hybridization despite pre- and post-pollination reproductive barriers between two *Silene* species. *AoB Plants, 8*, plw032. doi:10.1093/aobpla/plw032

Zhang, J. L., Zhang, C. Q., Gao, L. M., Yang, J. B., & Li, H. T. (2007). Natural hybridization origin of *Rhododendron agastum* (Ericaceae) in Yunnan, China: inferred from morphological and molecular evidence. *Journal of Plant Research, 120*(3), 457-463. doi:https://doi.org/10.1007/s10265-007-0076-1

Zhang, N. N., Yu, J. J., Wang, Y. H., & Gong, X. (2018). Molecular evidence for asymmetric hybridization in three closely related sympatric species. *AoB Plants, 10*(1), ply011. doi:http://dx.doi.org/10.1093/aobpla/ply011

Zhang, R., Gong, X., & Ryan, F. (2017). Evidence for continual hybridization rather than hybrid speciation between *Ligularia duciformis* and *L. paradoxa* (Asteraceae). *PeerJ, 5*, e3884. doi:http://dx.doi.org/10.7717/peerj.3884

Zheng, S. L., Tian, X. L., Huang, C. L., Wang, L. J., Feng, Y., & Zhang, J. L. (2017). Molecular and morphological evidence for natural hybridization between *Rhododendron decorum* and *R. delavayi* (Ericaceae). *Biodiversity Science, 25*(6), 627-637. doi:10.17520/biods.2017090

Zhu, X. F., Li, Y., Wu, G. L., Fang, Z. D., Li, Q. J., & Liu, J. Q. (2009). Molecular and morphological evidence for natural hybridization between *Primula secundiflora* Franchet and *P. poissonii* Franchet (Primulaceae). *Acta Biologica Cracoviensia Series Botanica, 51*, 29-36. doi:doi:10.1186/1471-2229-9-1
